# Benefits of Expressive Writing on Healthcare Workers’ Psychological Adjustment During the COVID-19 Pandemic

**DOI:** 10.3389/fpsyg.2021.624176

**Published:** 2021-02-25

**Authors:** Rossella Procaccia, Giulia Segre, Giancarlo Tamanza, Gian Mauro Manzoni

**Affiliations:** ^1^Faculty of Psychology, eCampus University, Novedrate, Italy; ^2^Department of Psychology, Catholic University of Milan, Milan, Italy

**Keywords:** COVID-19, healthcare workers, psychological adjustment, expressive writing, distress

## Abstract

COVID-19 outbroke in Wuhan, China, in December 2019 and promptly became a pandemic worldwide, endangering health and life but also causing mild-to-severe psychological distress to lots of people, including healthcare workers (HCWs). Several studies have already showed a high prevalence of depression, anxiety, and post-traumatic symptoms in HCWs but less is known about the efficacy of psychological interventions for relieving their mental distress. The aims of this study were: (1) to evaluate the psychological adjustment of Italian HCWs during the COVID-19 pandemic; (2) to investigate the efficacy of an expressive writing (EW) intervention, based on Pennebaker’s paradigmatic protocol, on their psychological adjustment; (3) to analyze if outcomes of EW vary in function of individual differences (age, gender, marital status, and baseline values of symptoms). Fifty-five HCWs were randomly assigned to one of two writing conditions: EW (*n* = 30) or neutral writing (NW; *n* = 25). Psychological adjustment (in terms of ptsd, depression and global psychopathology’s symptoms, perceived social support, and resilience) was assessed before and after three writing sessions. Participants who received the EW intervention showed higher improvements in ptsd, depression, and global psychopathology symptoms. Improvements in EW group varied in function of age, gender, marital status, and baseline values: young, men, married participants and those who had higher baseline scores showed a higher reduction of psychological distress symptoms while women, single and those who had lower baseline value showed increased social support, and resilience. In conclusion, the EW intervention had positive effects which varied in function of individual differences on HCWs’ psychological health.

## Introduction

Since December 2019, a pneumonia epidemic caused by the 2019 novel coronavirus (SARS-COV-2) outbroke in Wuhan and spread across China rapidly; it became a global pandemic within the following 2 months [World Health Organization (WHO), 2020]. Italy was, after China, the second in time country most affected by the COVID-19 outbreak.

Although higher levels of psychological distress have been reported among the general population (Serafini et al., 2020), healthcare professionals, given their crucial role in managing these emergency situations, seem to be more vulnerable. Overall, pandemic requires intense and prompt responses in terms of healthcare: healthcare workers (HCWs), either directly or indirectly, are involved in delivering care to patients, fighting at the frontline against the virus. Medical staff and affiliated HCWs are under both physical and psychological pressures. Considering that, at a normal time, nearly half of physicians report burnout, or emotional burden due to work-related stress (West et al., 2018), supporting their mental health in such an overwhelming COVID-19 sanitary emergency is a critical part of the public health response.

Self-reported psychological problems are prevalent in HCWs during the COVID-19 pandemic. A recent review (Preti et al., 2020) analyzed the effects of epidemic and pandemic outbreaks on HCW’s mental health: anxiety (45%), depression (27.5–50.7%), general psychiatric symptoms (17.3–75.3%), post-traumatic stress disorder (11–73.4%), insomnia (34–36.1%), and work-related stress symptoms (18.1–80.1%) are the most common symptoms. In particular, it has been stated that female healthcare professionals and nurses exhibited higher rates of affective symptoms compared to male and medical staff, respectively (Pappa et al., 2020).

Moreover, Chew et al. (2020) demonstrated a possible bi-directional association between the physical and psychological symptoms among HCWs during the COVID-19: timely psychological interventions for HCWs with physical symptoms should be considered, once an infection has been excluded.

Healthcare workers should be aware of the early signs of mental fatigue, avoiding those to affect their emotional well-being. Recent studies (Kinman et al., 2020; Polizzi et al., 2020) have shown the importance of individual coping strategies: acceptance, behavioral activation and mindfulness could foster resilience and recovery by increasing tolerance to distress, enhancing feelings of connectedness and support, and encouraging actions that are goal-directed and value-driven. Reduced morbidity has been associated with both practical and psychological support (Kisely et al., 2020; Pappa et al., 2020). Less is known about interventions to mitigate the emotional impact of epidemics on HCWs (Gold, 2020). Health care professionals could benefit from different resources such as helplines, online therapy and group counseling sessions to reduce anxiety, distress, and insomnia symptoms.

Although evidence-based effective interventions and treatments in the healthcare system and among healthcare providers are available, stigma and lack of time limit their uptake, even in normal times (Knaak et al., 2017).

Many barriers limit the implementation of conventional evidence-based interventions in this emergency situation. Not all HCWs are willing to receive psychological treatment, individually or as a group therapy (Chen et al., 2020).

Secondly, traditional face-to-face psychotherapy is not recommended during quarantine, switching most of the therapies to remote sessions.

Moreover, another issue that has arisen is that during this emergency situation people tend to experience a wide range of mental health problems, while evidence-based interventions usually focus on a single disorder (Yang et al., 2020).

People particularly benefit from confiding about traumas (Vrij et al., 2002). Disclosing information may allow people to release their mind from unwanted thoughts, help them to make sense of upsetting events and improve their emotion regulation, all of which can have positive consequences on mental and physical health (Frattaroli, 2006).

Expressive writing (EW) is a simple and straightforward exercise. The reference model is based on Pennebaker (2004), which states that expressing deeper thoughts and feelings can alleviate the individual’s physical and psychological health. Over the past 25 years (see Frattaroli, 2006; Pennebaker and Chung, 2007), several researchers have examined the effects of writing about traumatic life events. Pennebaker’s EW task involves writing about a traumatic experience for a controlled period of time (usually between 15 and 30 min), on consecutive days (usually from 2 to 4 days, Pennebaker, 1997). Although this technique has been compared to exposure-based therapies for posttraumatic stress disorder (Sloan et al., 2005, 2007), research on reducing posttraumatic stress symptoms through EW has shown inconsistent results (see Frisina et al., 2004). While some studies did not find strong links between posttraumatic stress symptoms and EW (Pennebaker and Chung, 2007), several studies have shown the benefits of writing across different sessions about personal experiences with stressful life-events. This procedure has been associated with the reduction of physical and mental symptoms both in clinical and normal simples (Pennebaker and Beall, 1986; Pennebaker and Francis, 1996; Smyth, 1998; Smyth et al., 1999). In addition, researchers have explored various individual difference indices to identify those subgroups for whom EW is most beneficial (Baikie and Wilhelm, 2005; Stickney, 2010). Smyth et al.’s (1999) meta-analysis found that it had a greater impact on males than on females. Results of other studies (Paez et al., 1999; Baikie, 2003; Solano et al., 2003) showed that EW is more beneficial for those high in alexithymia and high in dissociation. It is essential to understand the conditions under which EW works and how to maximize its benefits (Lu and Stanton, 2010). A recent study found that EW positively impacted on HCWs’ adaptive coping strategies and work relational communication satisfaction. Similarly, EW was found to be a useful tool for nurses in high-stress areas: coping strategies are vital to fight against burnout and depression (Sexton et al., 2009).

Starting from these considerations, the first study hypothesis (H1) is that Italian HCWs have high levels of psychological distress during the COVID-19 pandemic. The second study hypothesis (H2) is that the EW intervention is effective in reducing psychological distress in Italian HCWs. The third hypothesis (H3) is that the outcomes of the EW intervention vary in function of individual differences (age, gender, marital status, and baseline value).

## Materials and Methods

### Study Design

This study is a randomized and controlled trial with two conditions [EW vs neutral writing (NW)] and two repeated measurements (before and after the writing intervention).

### Participants

One hundred HCWs who worked in two hospitals settled respectively, in middle and south Italy were asked to participate to the study.

To be included in the study healthcare professional have to work 24 h a week continuously for at least 6 months in the same hospital and they have to work from the pandemic outbreak in the frontline with COVID-19 patients, specifically in COVID-19 Intensive Care Unit (ICU) or COVID-19 hospital ward. Professionals were excluded if they have been working in the same structure for shorter periods of time (less than 6 months), or were not directly working in COVID-19 wards.

Fifty-five out of them accepted to participate and were included in the study. Data were collected between April and June 2020. Participants were mainly females; the median age was 46.42 years old (SD = 9.9) and the majority were married or cohabiting in a stable way. Nurses comprised more than half of the sample, followed by physicians and allied HCWs. Majority had a degree (Table 1).

**TABLE 1:** Demographics.

**Total number**	**55**	
**Occupational status**		
Nurse	30	54.54%
Physicians	15	27.27%
Allied HCWs	10	18.18%
**Gender**		
Male	14	25.45%
Female	41	74.54%
**Marital status**		
Married or cohabiting	42	76.36%
Single	13	23.64%
**Age (years)**		
Mean (SD)	46.42 (9.9)	
Min-max	28	61
**Education**		
Degree	31	56.36%
Post-graduate degree	24	43.64%

#### Procedure

**FIGURE 1:**
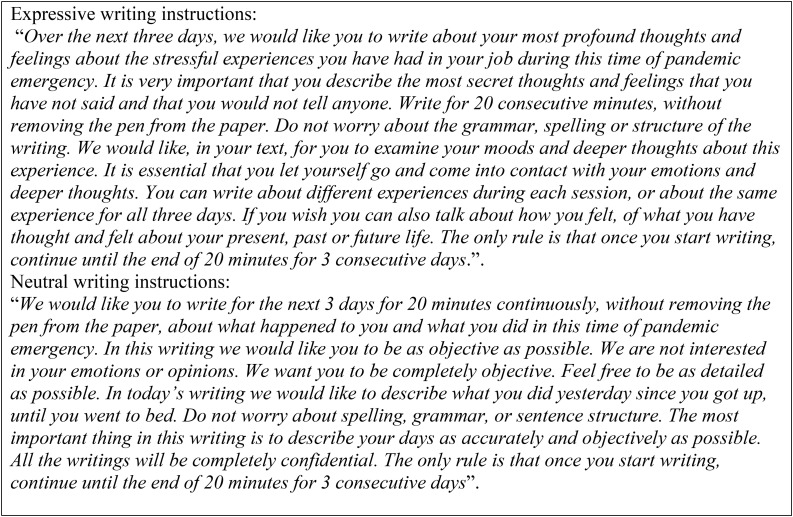
Expressive and neutral writing instructions.

Participants were randomly assigned to one of two writing conditions: EW (*n* = 30) or NW (*n* = 25). EW is a tool through which subjects describe their deepest thoughts and feelings about emotional events. NW is a comparison instrument, through which participants describe an event in a more objective way, without focusing on emotions, thoughts, or feelings (see Figure 1 for instruction).

This study was carried out in keeping with the Ethics Code of Italian Psychologists and approved by the Ethics Committee of e Campus University. Informed written consent was obtained from participants. The data were handled in keeping with General Data Protection Regulation (GDPR), Regulation UE 2016/679. All participants received an envelope including the information about the aims of the study, consent forms, a socio-demographic questionnaire, and all the other study questionnaires (Time 1). They completed them individually at home and then they received another envelope with writing instructions. Three days after filling in those questionnaires, participants were asked to write at their home for three consecutive days for 20 min each time according to the two writing conditions and, after 1 week, they were asked to fill in again the study questionnaires (Time 2). Literature has shown contradictory results for the spacing of disclosure sessions. Smyth (1998) conducted a meta-analysis and showed larger effect sizes in studies with weekly disclosure sessions (7 days intervals between each writing session) than studies with daily sessions; number and length of writing sessions were unrelated to improvement. On the contrary, no significant differences between daily and weekly treatment groups were found in a study which manipulated the spacing of disclosure sessions (Frattaroli, unpublished). For what concerns the amount of time dedicated to writing sessions, it has been reported (Frattaroli, 2006) that writing for longer than 15 min is more effective. The present study followed the standard EW protocol, in which participants are usually asked to spend 15–30 min writing for three to five consecutive days (Pennebaker, 1997).

### Measures

*Demographic characteristics*: Each participant was asked to indicate sex, age, marital/relationship status, level of education, years of practice, and role currently held.

*The Beck Depression Inventory* (BDI-II; Beck et al., 1996; Italian validation by Ghisi et al., 2006): The BDI-II was used to assess depressive symptoms. This measure includes 21 items, focused on cognitive, affective, motivational, and behavioral components of depression. For each item, this instrument uses a scale ranging from “0,” corresponding to a negative response (e.g., 0 = “I do not feel sad”), to “3,” positive response. Items are summed up to yield a total score. Each item was scored on a four-point scale, with a total score of 63. Based on the Italian validation, a cut-off score ≥12 identified the presence or the absence of depression. Scores were categorized as 13–19, mild depression; 20–28, moderate depression; and 29–63, severe depression. The Cronbach’s α coefficient in normative or clinical samples has ranged from 0.80 to 0.87 (Beck et al., 1996). In this study, the α coefficient was respectively, 0.82 at Time 1, and 0.83 at Time 2.

*Los Angeles Symptom Checklist* (LASC; King et al., 1995). The *LASC* is a self-report instrument. It includes 43 items and measures overall global distress related to trauma exposure, overall PTSD symptomology severity, and PTSD symptoms on three subscales (re-experiencing, avoidance/numbing, and hyperarousal). The instrument was shown to possess high internal consistency with α coefficients ranging from 0.88 to 0.95 (King et al., 1995). In this research α coefficients were 0.92 (Time 1) and 0.93 (Time 2). That’s there is not yet an Italian validation, LASC items were translated in Italian following back translation procedure.

*Symptom Check List – 90 Revised* (Derogatis, 1994; SCL-90: Italian version by Prunas et al., 2012). The SCL-90R is a 90 question self-report inventory that is made up of 90 items on disorders that may have been tried in the last week. Subjects give a rating from 0 (not at all) to 4 (very much) on a Likert scale. Items converge in 10 symptomatic subscales of different significance (somatization, obsessive-compulsive disorder, interpersonal sensitivity, depression, anxiety, hostility, phobic anxiety, paranoid ideation, psychoticism, and sleep disturbances). For each scale, the relative score is calculated as the average of the answers. A global index is also calculated (GSI-Global Score Index) as the average of all answers. Cronbach’s α coefficients higher than 0.70 were considered acceptable (Peterson, 1994). In this study, the α coefficient was 0.97 at Time 1 and at Time 2 both.

*Multidimensional Scale of Perceived Social Support* (MSPSS; Zimet et al., 1988; Italian validation by Prezza and Principato, 2002). The MSPSS is a self-report instrument; it includes 12 items that converge in three dimensions: family, friends, and significant others. Each item is rated on a seven-point Likert-type response format (1 = very strongly disagree; 7 = very strongly agree). A total score is calculated by summing up all the answers. The possible score range is between 12 and 84, the higher the score the higher the perceived social support. The possible score range for the subscales/dimensions is between 4 and 28. Any mean scale score ranging from 1 to 2.9 could be considered low support; a score of 3–5 could be considered moderate support; a score from 5.1 to 7 could be considered high support. Cronbach’s α coefficients range from 0.85 to 0.91 (Zimet et al., 1988). In this research α coefficients were 0.95 (Time 1) and 0.86 (Time 2).

*Resilience Scale for Adult* (RSA; Friborg et al., 2003; Italian validation by Di Fabio and Busoni, 2008). The RSA is a 33-items self-report instrument for evaluating six protective dimensions of resilience in adults: (1) perception of the self, (2) planned future, (3) social competence, (4) family cohesion, (5) social resources, and (6) structured style. Item-response ranges from one to seven and scores vary between 33 and 231, with higher scores indicating higher levels of resilience. Previous research showed Cronbach’s α from 0.67 to 0.81 and total score 0.88. In this study α coefficients were 0.87 at Time 1 and 0.89 at Time 2.

### Statistical Analysis

Descriptive analysis was carried out computing baseline values for every variable, considering total score and subscales to include a wide range of distress dimensions.

Specifically, we analyzed ptsd (reexperiencing, avoidance, and hyperarousal), depression and global psychopathology’s symptoms (Global Severity Index) (somatization, obsessive-compulsive disorder, interpersonal sensitivity, depression, anxiety, hostility, phobic anxiety, paranoid ideation, psychoticism, and sleep disturbances), perceived social support (significant other, family, and friend), and resilience.

Differently, since the small size of the sample, to improve the power of the statistics, we considered for hypothesis 2 and 3 only the total scores of each investigated variable (ptsd symptoms, depression symptoms, Global Severity Index, perceived social support, and resilience).

Repeated-measure ANOVAs were employed to test the effects of the EW intervention in comparison to NW on the study outcomes. All ANOVA models included a within-subject factor (pre scores and post scores), a between-subjects factor (EW vs NW) and their interaction, which was probed by means of plots in case of statistical significance. All ANOVA models also included the baseline value as a covariate variable, to control the effects of any significant differences in scores between EW and NW groups in pre-writing time.

Finally, delta values (Δ) were computed for the total scores as differences between pre-scores and post-scores, and were then regressed in EW group on age, gender (male-female), marital status (unmarried vs married or cohabiting), and baseline values in hierarchical multiple regression models. The SPSS 21 software was used.

## Results

### Psychological Conditions of Italian HCWs During the COVID-19 Pandemic

Baseline descriptive statistics (Table 2) show a high level of PTSD according to the LASC cut-off for the PTSD Severity Index (see King et al., 1995, p. 14) as well as high symptoms of hyperarousal, avoidance, and reexperiencing. A high level of psychopathology was also observed on the SCL 90R Global Severity Index, which resulted to be higher than the suggested cut-off (*T*-value ≥ 63; Derogatis, 1994). In particular, high scores were found in the somatization, depression, obsessive-compulsive disorder, anxiety, sleep disturbances, and interpersonal sensitivity scales, while lower scores were found in the phobic anxiety, paranoid ideation psychoticism and hostility scales. With respect to depression symptoms assessed through the BDI II (Table 3), 45.45% of participants were in the minimal range, 32.73% in the mild depression range, 10.91% in the moderate depression range and 10.91% in the severe depression range. Participants perceived a moderate level of total social support according to Zimet et al. (1988). In sub-scales, high levels of perceived support from significant others and from family were observed, while a moderate level of perceived support from friends was found. Finally, they showed moderate level of resilience, according to Di Fabio and Busoni (2008).

**TABLE 2:** Baseline descriptive statistics of psychological variables in the whole samples.

**Variable**	***N***	**Mean**	**Standard deviation**	**Minimum**	**Maximum**
Reexperiencing	55	4.04	2.08	1	7
Avoidance	55	5.4	2.22	3	10
Hyperarousal	55	11.53	5.34	5	23
Ptsd	55	20.96	7.97	10	36
Depression (BDI-II)	55	16.36	9.78	5	45
Somatization	55	15.84	9.78	1	37
Obsessive-compulsive disorder	55	10.33	8.11	1	28
Interpersonal sensitivity	55	4	2.61	0	9
Depression (SCL90R)	55	14.84	10.23	4	35
Anxiety	55	9.67	9.91	2	31
Hostility	55	2.33	1.93	0	7
Phobic anxiety	55	3.56	5.88	0	17
Paranoid ideation	55	3.47	2.38	0	10
Psychoticism	55	3.18	2.69	0	12
Sleep disturbances	55	5.58	4.49	0	12
GSI	55	75.13	51.42	19	172
Significant others	55	5.14	1.14	1	6
Family	55	5.09	0.90	1	6
Friend	55	3.98	0.93	1	6
Support	55	4.74	0.89	1	6
Resilience	55	116.29	10.53	95	144

**TABLE 3:** Depression scores distribution.

**Depression level**	***N***	**Frequency percent**
Minimal range	25	45.45
Mild depression	18	32.73
Moderate depression	6	10.91
Severe depression	6	10.91

### The EW Effects

**FIGURE 2:**
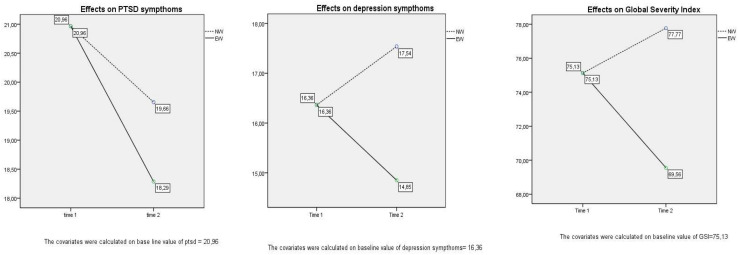
Graphs of repeated-measure ANOVAs.

Statistically significant interaction effects were found for ptsd symptoms, depression symptoms, and Global Severity Index. No effects for social support and resilience were found (see Figure 2 and Table 4).

**TABLE 4:** Repeated-measure ANOVAs.

	**Sum of square**	**df**	**Mean square**	***F***	***p***
ptsd	1.553	1	1.553	0.282	0.598
Ptsd × ptsd effect	6.779	1	6.779	1.231	0.272
Ptsd × writing condition	12.777	1	12.777	13.725	0.002
Depression	3.106	1	3.106	0.412	0.521
Depression × depression effect	5.842	1	5.842	0.775	0.383
Depression × writing condition	38.679	1	38.679	6.123	0.028
GSI	93.066	1	93.066	0.928	0.34
GSI × GSI effect	235.021	1	235.021	2.343	0.132
GSI × writing condition	335.135	1	335.135	5.232	0.03
Social support	72.948	1	72.948	3.425	0.56
Social support × social support effect	25.615	1	25.615	2.074	0.592
Social support × writing condition	2.33	1	2.33	0.116	0.735
Resilience	0.928	1	0.928	0.116	0.735
Resilience × resilience effect	2.716	1	2.716	0.338	0.563
Resilience × writing condition	0.965	1	0.965	0.12	0.73

Plots showed that: (1) ptsd symptoms reduced significatively only in EW group (ptsd × writing condition *F* = 13.725, *p* = 0.002) (2) depression symptoms reduced in EW group while it increased in NW group (depression × writing condition: *F* = 6.123, *p* = 0.02); (3) the SCL-90R Global Severity Index reduced in EW group, while it increased in the NW group (GSI × writing condition: *F* = 5.232; *p* = 0.03).

### Predictors of Changes

Multiple regression analyses were then performed in the EW group with Δ values of ptsd symptoms, depression symptoms, Global Index Severity, perceived social support, and resilience entered as dependent variables and age, gender, marital status, and baseline values as predictors. Results (Table 5) show that change in ptsd symptoms is predicted firstly by marital status and then by baseline value. In particular, married participants and the ones who presented higher levels of ptsd symptoms before writing sessions showed a higher improvement in post-traumatic reaction after EW.

**TABLE 5:** Multiple regression analyses in EW group (Δ values as “dependent variables”; age, gender, marital status, and baseline value as “predictors”).

**Criterion**	**Predictors**	**β**	***T***	**Sig**nificant	***R*-square**
Δ ptsd symptoms	Age	–0.124	–0.717	0.48	0.261
	Gender	–0.3	–1.69	0.103	
	Marital status	0.444	2.531	0.018	
	ptsd t1	0.163	1.938	0.047	
Δ depression symptoms	Age	–0.355	–2.179	0.039	0.375
	Gender	–0.322	–1.938	0.064	
	Marital status	0.439	2.66	0.013	
	Depression t1	0.068	0.417	0.681	
Δ GSI	Age	–0.432	–2.65	0.014	0.355
	Gender	–0.374	–2.205	0.037	
	Marital status	0.257	1.566	0.013	
	GSI t1	–0.363	–2.17	0.04	
Δ social support	Age	–0.069	–0.994	0.33	0.884
	Gender	0.148	2.113	0.045	
	Marital status	–0.215	–2.637	0.014	
	Social support t1	–1.024	–12.712	0.0001	
Δ resilience	Age	0.242	1.455	0.158	0.316
	Gender	0.015	0.086	0.932	
	Marital status	–0.515	–3.015	0.006	
	Resilience t1	0.216	1.22	0.234	

Depression symptoms were predicted by marital status and age. Young and married participants’ depression levels improved more after the writing intervention.

Age, gender, and baseline value predicted change in global psychopathology, with young, men and those who showed higher GSI score at baseline had higher improvements after EW.

Social support is predicted by gender, marital status, and baseline value: women, single and the ones who presented lower levels of perceived social support before writing sessions showed a higher improvement. In the same direction, resilience is predicted by marital status with higher improvement in not married participants.

## Discussion

The aims of the study were to evaluate the psychological adjustment of Italian HCWs during the COVID-19 pandemic and to investigate the efficacy of an EW intervention to improve their mental well-being. The effects variability in function of individual differences was also investigated.

As regards the first aim, our findings mirror the trend in previous studies on the psychological impact of the COVID-19 affection among the general population in China during its initial stages (Kang et al., 2020; Li et al., 2020; Wang et al., 2020). Specifically, high level of global distress, with severe symptom of somatization, anxiety, obsessive compulsive disorder, sleep disturbances, and specific post-traumatic reactions (reexperiencing, avoidance, and hyperarousal) were found in our sample. Results confirm data from previous pandemics that underlined how HCWs might experience acute stress reactions, particularly after quarantine, developing symptoms of post-traumatic stress disorder, depression, and anxiety (Gold, 2020; Kinman et al., 2020; Pappa et al., 2020). Previous researches had found that psychosomatic symptoms (such as somatization) could accompany specific physical manifestations of various diseases, due to the psychological sequelae of the pandemic outbreaks (Chew et al., 2020; Xiang et al., 2020).

It is widely recognized that HCWs are need of psychological support interventions to help them to mitigate the effects of the COVID-19 pandemic on their well-being in short and long time. In particular, they are in need of recognizing and elaborating emotional stress and pain in order to avoid that unelaborated pain can become chronic and cumulative, with important personal and professional implications (Kinman et al., 2020).

For what is concerned to the second study hypothesis, our data confirm the efficacy of EW, in promoting the reflection upon stressful events and the elaboration of negative feelings that may over the time overwhelm the person’s ability to cope with emotional distress, according to previous research (Tonarelli et al., 2017). A significant reduction in several symptoms were found in EW group, while NW group did not show improvement or even presented increased scores in clinical dimensions, maybe due to the continuation of the stress associated with the emergency.

In particular, the study results support the hypothesis that focusing on emotions, feelings, and deeper thoughts allow HCWs to reduce various distress symptoms, such as ptsd symptoms. It impacts positively also on depression symptoms and global psychopathology according to the previous researches (Greenberg et al., 1996; Schoutrop et al., 1997, 2002; Sloan and Marx, 2004a,b).

As regard the third hypothesis, regression analysis showed the moderating role of individual differences in EW benefits. Previous researchers have, in fact, explored different individual variables to identify subgroups for whom EW is more beneficial (Lu and Stanton, 2010). In this study, baseline value of ptsd symptoms predicts the change in post intervention scores: participants who reported more severe symptoms before the writing showed higher benefits, according to previous research (Di Blasio et al., 2015). It should be noted that since the HCWs in this study were part of a normative group, the ptsd, depression, and global psychopathology’s symptoms do not have clinical significance but indicate sub-clinical symptoms. Because some research (Brugha et al., 2011; Furukawa et al., 2012) noted that without intervention sub-clinical symptoms tend to increase, the results suggested that the EW intervention in the normative group could be useful to buffer the negative development of psychological distress.

Gender effects were also found in this study, with men showing higher benefits in global psychopathology symptoms and women presenting higher level of perceived social support after EW.

Previous research has underlined gender differences in EW efficacy, but the results are still inconsistent. Some authors stated that men showed higher benefits, but other studies found no difference in outcomes between men and women, and among the studies that did, there is nearly an equal number supporting the argument that the benefits are stronger for women (Stickney, 2010). For example, Smyth et al.’s (1999) meta-analysis suggested that studies with a higher percentage of men had larger effect sizes (i.e., better outcomes) than studies with more women, but Frattaroli’s (2006) found no such effect.

Our findings suggested that EW’s efficacy in reducing psychopathological symptoms is higher in men. According to Range and Jenkins (2010), we suppose that men tend more to inhibit emotional expression and, when they are “forced” to focus on emotions and feelings, they benefit more than women, who are more used to expressing and verbalizing emotions. On the contrary women showed increased scores in perceived social support after writing and we presume that it’s because when women are asked to communicate about negative emotion and thoughts, they perceived the task as and index of closeness and support.

Finally, marital status and age resulted to predict changes in outcome variables, with younger and married participants showing higher benefits, except for social support and resilience that increased more in single people. Authors of previous studies suggested that staff who were younger (Nickell et al., 2004; Sim et al., 2004; Tam et al., 2004; Wu et al., 2009; Austria-Corrales et al., 2011), or parents of dependent children (Maunder et al., 2004; Koh et al., 2005) are more vulnerable to psychological distress, probably because they are afraid of bringing the virus to their home and, in addition, they do not want their families to worry about them (Chen et al., 2020). HCWs may also feel the inner conflict between their desire to care for patients and, at the same time, their need to protect themselves and their loved ones from the life-threatening infection (Kisely et al., 2020).

On the contrary, higher improvements in perceived social support and in resilience in not married participant were found. We presume that single participants could have less opportunity to communicate their inner feeling during the crisis, so they could benefit more from the procedure because they live the research like an opportunity to report and reflect on their feelings and negative emotion.

## Conclusion

In conclusion, although psychological distress in HCWs is common in situations where they are under pressure to care many potentially infectious patients, EW can help to mitigate it. This kind of intervention could maximize the internal resources of HCWs by effectively improving their quality of life and, consequently, also patient outcomes. The development of a coherent narrative could help them to reorganize and elaborate the traumatic memories, allowing the structuration of more adaptive internal schemas.

The strength of the EW is the rapidity with which it allows the remission of symptoms and the expression of feelings. However, the impact of individual differences highlights the need to accompany this tool with long-term intervention, which could also benefit those who need a deeper elaboration of negative emotion.

The results are interesting but there is some limitation. The most important study limitation is the small sample size, which limited the statistical power of tests and restricts the generalization of results. For that we analyzed in hypothesis 2 and 3 only total score of global dimensions (ptsd symptoms, depression symptoms, GSI, social support, and resilience) but it could be interesting to consider all the sub-symptoms to deeply understand the effect of distress.

In addition, lack a follow-up testing after a longer period (6–12 months) that could allow to better understand if the changes in psychological adjustment are consistent and stable during the time.

Finally, to better understand the process of elaboration allowed by the writing intervention, the quantitative analysis could be successfully accompanied by a qualitative analysis of the writing to identify the emotional changes, the narrative markers of the inner process of meaning making, to detect the coping strategies and the changes of thematic content across 3 days (Tonarelli et al., 2017).

## Data Availability Statement

The raw data supporting the conclusions of this article will be made available by the authors, without undue reservation.

## Ethics Statement

The studies involving human participants were reviewed and approved by the E Campus University Ethic Committee. The patients/participants provided their written informed consent to participate in this study.

## Author Contributions

All authors listed have made a substantial, direct and intellectual contribution to the work, and approved it for publication.

## Conflict of Interest

The authors declare that the research was conducted in the absence of any commercial or financial relationships that could be construed as a potential conflict of interest.
